# Recurrent visual and narrative motifs as indicators of stable neurocognitive patterns: evidence from three clinical cases

**DOI:** 10.1192/j.eurpsy.2026.11460

**Published:** 2026-07-31

**Authors:** P. A. Caldas

**Affiliations:** Psychiatry, Hospital “Dr. Rodríguez Lafora”, Madrid, Spain

## Abstract

**Introduction:**

This case series examines visual (drawings) and narrative productions as potential expressions of structural and functional regularities in brain–mind organization. The material was interpreted in the light of contemporary theoretical frameworks that consider the existence of neuronal predispositions and innate behavioral patterns. As a historical reference, these productions were compared with a 1550 engraving, revealing striking similarities in morphology and explicit meaning. The findings suggest the presence of recurrent expressive motifs that may reflect stable architectures of neural networks and functional organization of the mind. Human mental life appears to be shaped by deep structural and functional regularities, which can manifest through expressive productions. Recent proposals in cognitive neuroscience suggest the existence of phylogenetically shaped “neuronal preparedness states” that predispose the emergence of specific motifs in behavior and language. In parallel, classical psychological and ethological theories posit that innate behavioral patterns reveal relatively stable internal organizations in interaction with environmental signals. The analysis of spontaneous visual and narrative productions provides a framework to explore the convergence of these perspectives and the appearance of recurrent motifs.

**Objectives:**

To examine visual and narrative motifs in three clinical cases and assess their correspondence with each other and with analogous historical material, as potential indicators of stable structural and functional brain–mind patterns. To promote further research in this field.

**Methods:**

Visual and narrative material was occasionally collected from three patients in diverse clinical contexts (private psychotherapy and chronic hospital services across two continents, over more than 20 years). Two patients were asked to produce free drawings, while the narrative material consisted of spontaneous written production. A descriptive comparison of formal and semantic aspects was carried out between the clinical materials and a 1550 engraving.

**Results:**

Across the three patients —unrelated to each other and going through a crisis (two chronic psychoses and one pubertal disruption)— the Sun/Moon/Star motif emerged spontaneously in a precise configuration, without external influences suggesting such content. Comparisons of drawings, narratives, and the historical engraving revealed a high degree of formal and thematic similarity.

**Conclusions:**

These cases suggest that visual and narrative productions may reflect stable neurocognitive patterns, likely of innate origin. The recurrence of motifs across individuals, historical periods, and cultures highlights the need for further research, incorporating diferent kinds of motif analysis, larger samples, and developmental longitudinal studies.

**Disclosure of Interest:**

None Declared

